# Screening Discriminating SNPs for Chinese Indigenous Pig Breeds Identification Using a Random Forests Algorithm

**DOI:** 10.3390/genes13122207

**Published:** 2022-11-25

**Authors:** Jun Gao, Lingwei Sun, Shushan Zhang, Jiehuan Xu, Mengqian He, Defu Zhang, Caifeng Wu, Jianjun Dai

**Affiliations:** 1 Institute of Animal Husbandry and Veterinary Science, Shanghai Academy of Agricultural Sciences, Shanghai 201106, China; 2 Key Laboratory of Livestock and Poultry Resources (Pig) Evaluation and Utilization, Ministry of Agriculture and Rural Affairs, Shanghai 201106, China; 3 Shanghai Municipal Key Laboratory of Agri-Genetics and Breeding, Shanghai 201106, China; 4 Shanghai Engineering Research Center of Pig Breeding, Shanghai 201106, China

**Keywords:** breed identification, single-nucleotide polymorphisms (SNP), random forests

## Abstract

Chinese indigenous pig breeds have unique genetic characteristics and a rich diversity; however, effective breed identification methods have not yet been well established. In this study, a genotype file of 62,822 single-nucleotide polymorphisms (SNPs), which were obtained from 1059 individuals of 18 Chinese indigenous pig breeds and 5 cosmopolitan breeds, were used to screen the discriminating SNPs for pig breed identification. After linkage disequilibrium (LD) pruning filtering, this study excluded 396 SNPs on non-constant chromosomes and retained 20.92~−27.84% of SNPs for each of the 18 autosomes, leaving a total of 14,823 SNPs. The principal component analysis (PCA) showed the largest differences between cosmopolitan and Chinese pig breeds (PC1 = 10.452%), while relatively small differences were found among the 18 indigenous pig breeds from the Yangtze River Delta region of China. Next, a random forest (RF) algorithm was used to filter these SNPs and obtain the optimal number of decision trees (ntree = 1000) using corresponding out-of-bag (OOB) error rates. By comparing two different SNP ranking methods in the RF analysis, the mean decreasing accuracy (MDA) and mean decreasing Gini index (MDG), the effects of panels with different numbers of SNPs on the assignment accuracy, and the statistics of SNP distribution on each chromosome in the panels, a panel of 1000 of the most breed-discriminative tagged SNPs were finally selected based on the MDA screening method. A high accuracy (>99.3%) was obtained by the breed prediction of 318 samples in the RF test set; thus, a machine learning classification method was established for the multi-breed identification of Chinese indigenous pigs based on a low-density panel of SNPs.

## 1. Introduction

Over thousands of years of domestication and natural selection, China has developed rich swine genetic resources, which not only promote the development of the Chinese swine industry, but also contribute to the improvement of pig breeds worldwide. There are many indigenous pig breeds in China; according to statistics, there are about 108 local pig breeds or strains. For example, the Taihu pig, known for its high fertility, can be divided into seven breeds based on its unique characteristics or traits [1]. However, due to a small population size, some breeds are gradually inbreeding and becoming degraded or even extinct. At the same time, another problem is the disorderly crossbreeding between different breeds, which makes it difficult to differentiate the genetic background of these individuals, and it is difficult to make an accurate identification by phenotypic observation. It is crucial to develop effective genetic testing tools for the accurate identification and conservation of these indigenous pig breeds. Moreover, the accurate identification of breeds of animals and their products has important practical applications, including the effective management of livestock genetic resources, assessing the level of crossbreeding between populations, refining breeding strategies and programs, and providing brand certification of certain breed-specific products [2]. Single-nucleotide polymorphisms (SNPs) as the most common type of genetic variation have many important applications in livestock, including genomic characterization studies, population diversity analysis, and pedigree identification [3,4,5].

The random forest (RF) algorithm is a machine learning method used for classification and regression, where each tree independently depends on the values of a random sampled vector [6]. RF draws bootstrap samples from the original data. For each of the bootstrap samples, an unpruned classification or regression tree is grown. Finally, new data are predicted by aggregating the predictions of the trees. At each bootstrap iteration, a tree grown using bootstrap samples to predict data that are not in the bootstrap samples yields an estimate of the “out-of-bag” (OOB) error rate [7]. RF can rank predictors based on correlations in classification rules. It provides two relevance measures: the mean decrease accuracy (MDA) and mean decrease Gini index (MDG). The MDA indicates the degree of decrease in the prediction accuracy of the RF, and the MDG calculates the effect of each variable on the heterogeneity of observations at each node of the classification tree [8]. Therefore, the higher the value of both, the higher the importance of the variables. When SNPs in genomic data are used as predictors in classification, these two ranking rules can be applied to the SNPs in the dataset, thus filtering out the important panel of discriminatory SNPs [2,9].

RF has been successfully applied to several livestock breed identification studies in recent years. Schiavo et al. [10] analyzed the PorcineSNP60 BeadChip array genotyping data of more than 2700 individuals from seven Italian pig breeds (three cosmopolitan-derived breeds and four autochthonous breeds) by an RF approach and correctly assigned breeds with an accuracy of close to 100% in the reduced SNP panel. However, to our knowledge, the application of RF to screening breed discriminating markers of Chinese pig breeds has not been reported yet. This study aims to develop low-density SNP panel for Chinese indigenous pig identification, and apply them to the genetic conservation, diversity analysis, and product traceability certification of breeds.

## 2. Materials and Methods

### 2.1. Data Information

The data analyzed in this study were derived from a 62,822 SNP genotype file for 24 pig breeds, including 159 individuals from 5 cosmopolitan breeds and 910 individuals from 19 indigenous breeds in the Yangtze River region of China. The data were generated by Zhao et al. [1] and are publicly available at the website (https://jbox.sjtu.edu.cn/l/uoaCjx/, accessed on 6 June 2022). In order to keep the number of individuals involved in the analysis above 20 for each Chinese indigenous pig breed, we removed the data from the genotype file for one breed (Dongchuan pigs, DC) because it contained only 10 individuals. Therefore, subsequent analysis based on a genotype file of 1059 individuals from 23 breeds (Table 1) was performed.

In the genotype file, 0 represents homozygotes with major allele (AA), 1 represents heterozygous (AB) and 2 represents homozygous with the rare allele (BB). The missing genotype data have previously been imputed using Beagle (version 5.0) software (Seattle-WA-USA) [11] and filtered by minor allele frequency (MAF) ≥ 0.05 [1].

### 2.2. LD Pruning

The “SNPrelate” package [12] in R program version 4.2.1 (Vienna-Austria) [13] was used to estimate LD squared allele frequency correlation (r2) for all pairwise comparisons between SNPs on the same chromosome with a sliding window of 500,000 base pairs and LD threshold of 0.2. After LD pruning, the retained SNPs on each chromosome were obtained and subsequently analyzed by principal component analysis (PCA), which was performed and visualized by Majorbio Cloud bioinformatic platform (https://cloud.majorbio.com/page/tools/, accessed on 16 July 2022) [14].

### 2.3. Random Forest Analysis

Random forest (RF) analysis was applied to the retained SNPs after LD pruning filtering using the random forest package in R [7]. The appropriate number of decision trees used in the RF classifier is estimated by the number of decision trees corresponding to the out-of-bag (OOB) error rate. Next, the mean decrease accuracy (MDA) and the mean decrease Gini index (MDG) in RF were used to rank all SNPs importance from highest to lowest, respectively.

The SNP panels consisting of the top 1000 and top 100 SNPs with the highest scores for both methods (MDA and MDG) were tested, and genotype data for the SNP panels were extracted from the original genotype dataset (1059 individuals from 23 breeds). The correlation between the number of SNPs and the error rate in the RF model were tested using 10-fold cross-validation of 5 replicates.

The newly generated genotype datasets were randomly, at a ratio of 7:3, formed into a training set (741 individuals of 23 breeds) and a test set (318 individuals of 23 breeds) in the RF model for further classification comparison. By comparing the accuracy of different SNP panels for breed classification and the coverage of SNP distribution on chromosomes, the suitable SNP panel were finally determined and their distribution of physical locations on the pig genome (Sscrofa11.1) [15] were plotted by CMplot package [16] in R program.

## 3. Results

### 3.1. LD Pruning 

Prior to the principal component analysis (PCA), LD pruning was performed for the SNPs in the delineated window, thereby removing interrelated SNPs to prevent the excessive variation in high LD regions from affecting the results. In this study, LD pruning filtering excluded 396 SNPs on non-constant chromosomes and retained 20.92–27.84% of SNPs on each of the 18 autosomes, leaving a total of 14,823 SNPs. The 1059 individual genotype files for these 14,823 SNPs were extracted from the original 23 breed genotype data and are available in Supplementary Table S1.

### 3.2. Principal Component Analysis (PCA)

**Figure 1:**
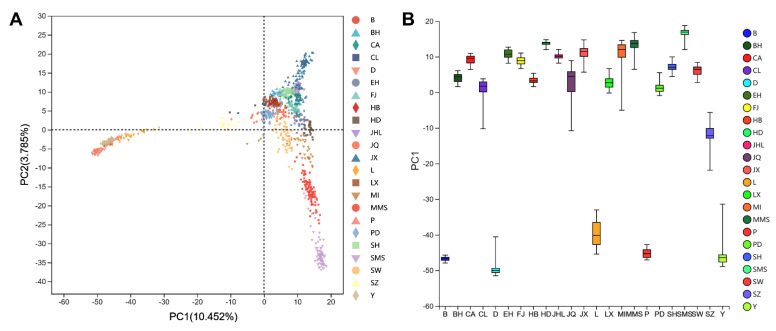
Principal component analysis (PCA) for 23 pig breeds based on 14,823 SNPs. (**A**) Plots of PC1 and PC2; (**B**) PC1 reflects the main difference between the cosmopolitan breeds and Chinese indigenous pig breeds.

The PCA’s first principal component (PC1) can explain 10.452% of the total variation, and the second principal component (PC2) can explain 3.785% of the total variation (Figure 1A). The PC1 reflects the main difference between five Western pig breeds (Duroc, Landrace, Yorkshire, Pietrain, and Berkshire) and Chinese indigenous pig breeds (Figure 1B), while relatively small differences were detected among the 18 Chinese indigenous pig breeds.

### 3.3. The Number of Decision Trees in RF Model

**Figure 2:**
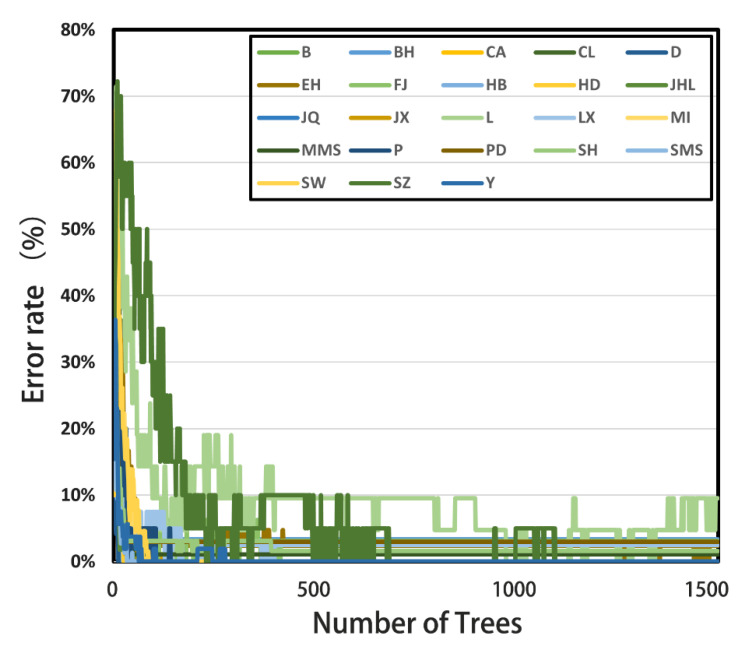
Correlation between the number of decision trees and out-of-bag (OOB) error rate in random forest model.The *X*-axis represents the number of decision trees used in the random forests, the *Y*-axis represents the out-of-bag (OOB) error rate, and the different colors in the legend indicate the 23 different pig breeds in this study.

The number of decision trees in the classifier is an important parameter for the classification correctness. It was shown that the error rates (OOB) were 1.04%, 0.76%, and 0.94% when the numbers of trees were 500, 1000, and 1500, respectively (Figure 2). Therefore, the subsequent decision tree parameter of this study was set to 1000.

### 3.4. The Most Discriminating SNPs

**Figure 3:**
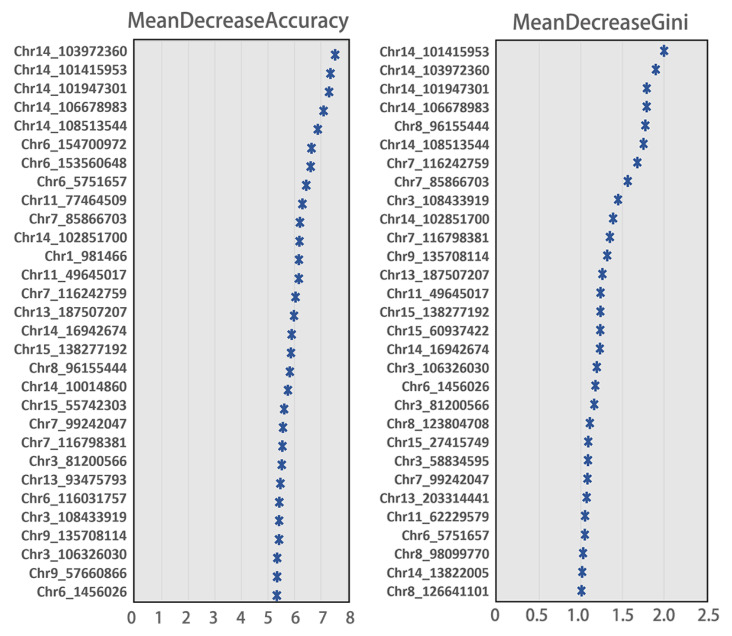
Top 30 important SNPs with the highest scores in random forest analysis.

The aim of this study was to select the most discriminating SNPs from 62K SNPs in pig genomes that could be used for breed identification. After LD pruning, we reduced the number of SNPs to 14,823. By RF analysis, we scored all the remaining 14,823 SNPs by MDA and MDG methods, and by ranking the scores from highest to lowest, we were able to generate the most discriminating SNP panel for breed identification. We counted the top 1000 SNPs ranked by these two methods, of which 681 were overlapping, indicating that the SNPs selected by these two methods are relatively consistent. Among the 30 SNPs with the highest scores selected by these two methods, several SNPs on chromosome 14 were ranked as high (Figure 3).

The *X*-axis represents the scores from the MDA or MDG measures and each row on the *Y*-axis represents an SNP, labeled by the chromosome and physical location.

### 3.5. Correlation between the Number of SNPs and Error Rate

**Figure 4:**
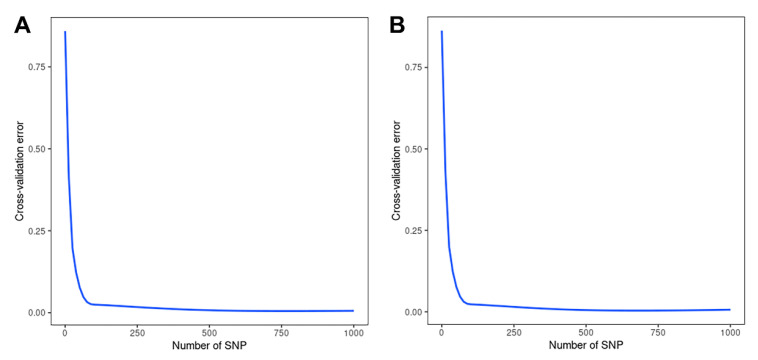
10-fold cross-validation with 1K SNP panel in RF analysis. (**A**) 1K SNP panel selected by MDA; (**B**) 1K SNP panel selected by MDG.

Using cross-validation, we compared the correlation between the 1000 SNPs selected for high information content and the cross-validation error rate and showed that the highest information content of 100 or more SNPs was able to significantly reduce the classification error rate. Additionally, when 500–1000 SNPs were used, the basic error rate was maintained at an extremely low level (Figure 4).

### 3.6. Classification Evaluation of a Panel of 1000 SNPs and 100 SNPs

We compared the errors of RF classification with 1000 SNPs (1K SNPs), each selected by MDA and MDG. The Mtry parameter is 31, which indicates the number of variables randomly sampled as candidates at each split. The 1K SNP panel selected by MDA measure for classification showed that five samples were inconsistent with expectations (5/1059) with an error rate of 0.47%, while the classification with a panel of 1K SNPs selected by MDG measure shows that six samples (6/1059) were inconsistent with expectations with an error rate of 0.57% The most 1K discriminating SNPs selected by MDA and MDG measures are listed in the Supplementary Table S2.

Next, we tested the effects of the 100 SNP panels, although the 100 most informative panels also provided a classification result with a relatively modest average error rate (43/1059 = 4.06%). However, its error rate was concentrated in only three breeds (L: 48%, P: 25%, SZ: 65%), as shown in Supplementary Table S3.

**Figure 5:**
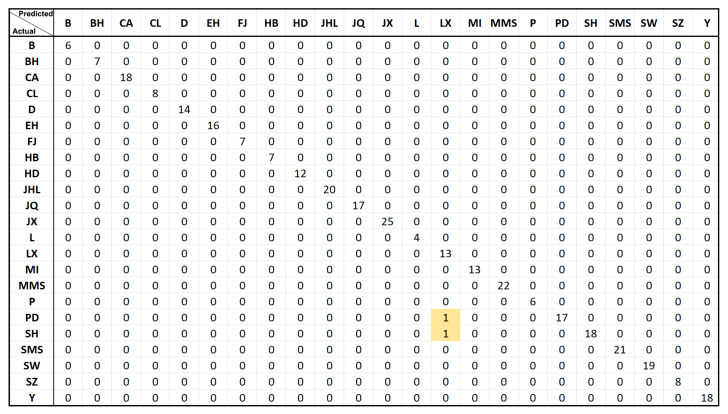
Validation of the established random forest model using the test set. The first header row shows the breeds predicted by the model for the classification of the 318 individuals in the RF test set, and the first column shows the actual observed classification of the breeds for the 318 individuals. The results show that, in each of the PD and SH breed, only one sample classification is inconsistent with the actual observed results (marked with a yellow background).

By comparing the error rates of the two 1K SNP panels, we finally chose the 1K SNP panel of MDA, and tested the breed classification of the RF model for the training set (741 samples) and the test set (318 samples), in which all the samples in the training set were correctly classified, while only 2 samples (No. 731 PD and No. 785 SH) in the test set were inconsistent with the actual observed breed results (2/318 = 0.0063, correct rate > 99.3%, Figure 5).

**Figure 6:**
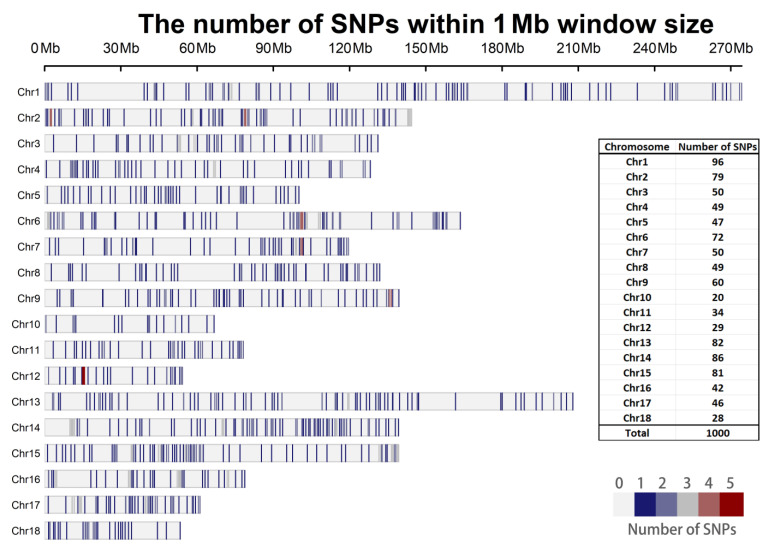
The distribution of 1K SNP panel selected by MDA method on the pig genome. The corresponding pig reference genome is Sscrofa11.1 and the number of SNPs on each chromosome is counted in the table on the right side of the picture.

On the other hand, we counted the distribution of SNPs on chromosomes in the 1K SNP panel and the 100 SNP panel by the MDA measure selected, where the 1K panel had a better coverage on chromosomes. For example, it has at least 20 SNPs on chromosome 10 (Figure 6). On the contrary, the 100 SNPs, due to the substantially reduced number, have no SNPs on chromosomes 10 and 16. Only 1–2 SNPs were retained on chromosomes 4, 5, 12, and 18. Therefore, we believe that too few SNPs, although able to reduce genotyping costs, leads to a decrease in accuracy and genomic coverage, especially when there are more breeds to be identified.

## 4. Discussion

Most of the 18 indigenous pig breeds in the Yangtze River region of China that we chose for this study are slow-growing and inferior to cosmopolitan breeds in terms of feed conversion and growth performance. However, due to their juicy, refreshing taste and tender quality, they are often widely preferred by consumers and their prices are much higher than those of ordinary commercial pigs. For example, two breeds in this study, Chunan (CA) and Jinhualiangtouwu (JHL), have long been used to produce high-quality cured ham and bacon [1]. Therefore, screening discriminating SNPs for the identification of indigenous pig breeds is not only useful for the conservation evaluation of these pig breeds, but it also provides a technical approach to certifying the products or derivatives of these unique breeds.

SNPs have been widely used for the breed assignment, product authentication, and pedigree identification of domestic animals, with the development of animal gene chips and high-throughput sequencing technologies. Ramos et al. obtained a total of 193 breed-specific SNPs in five pig breeds, which were used to assign an additional 490 individuals from the same breeds, showing that >99% of animals were correctly assigned, and demonstrating the high utility of breed-specific markers for breed assignment and traceability [17]. Several other approaches, including delta statistics, the fixation index (*F*_ST_), principal component analysis (PCA), and machine learning algorithms have been proposed to identify informative markers in livestock and poultry populations [2,18,19,20,21]. All of these methods had a high accuracy in breed assignments. For example, Seo et al. [20] used the RF model to select 44 SNPs as the minimum number of markers to classify 283 chicken individuals from 20 populations, and the accuracy reached up to 98.0%. However, when the number of breeds reaches dozens or more, or when there are relatively close genetic evolutionary relationships among some local breeds, a moderate increase in the number of highly informative SNPs may be more necessary to accurately identify and assign these breeds, although this may increase the cost of genotyping.

There are often many linkage disequilibrium relationships between SNPs due to the genotypes obtained from the high-density SNP arrays. These SNPs do not add information and even reduce the accuracy of estimation. Therefore, in the first step of this study, we filtered out about 80% of the linkage disequilibrium loci on each autosome from the original set of SNPs by LD pruning, and only retained SNPs that were not high in LD. The PCA results of the data of SNPs that underwent LD pruning showed the most significant differences between the five cosmopolitan pig breeds and the 18 Chinese indigenous pig breeds (PC1 = 10.452%), which was also consistent with what was expected from the actual situation. Although indigenous breeds experienced a long-term natural and artificial selection, these breeds may have a common ancestral origin or relatively close kinship, and thus cannot be effectively distinguished on the PCA plot. In contrast, the low-density SNP panel screened by the RF method allowed us to identify these indigenous pig breeds with accurate assignment.

Another issue is how many SNPs are appropriate for the identification of breeds. Too many SNPs will lead to increased typing costs and too few SNPs are likely to reduce the accuracy of classification results. In this study, we compared the classification results using 100 and 1K SNPs with the highest MDA score, and the results show that when 100 SNPs were selected, about 96% classification accuracy could be achieved, but the problem is that the error rate concentrates on few specific breeds. In contrast, using the 1K SNP panel improves the classification accuracy in terms of the results of training and test set classification. On the other hand, in terms of overall genome coverage, the 1K panel is essentially guaranteed to have more than 20 SNP markers on each autosome, which is more suitable as a tool for genetic quality monitoring of breeds at a genome-wide level.

## 5. Conclusions

In this study, genotype data of 1059 individuals from 23 pig breeds were used to screen a panel of 1K SNPs that could be used for Chinese indigenous pig breeds assignment by using linkage disequilibrium filtering, PCA analysis, and an RF algorithm. By optimizing the number of decision trees, comparing the panel of SNPs selected by MDA and MDG measures, and the correlation between the number of SNPs in the panel and the OOB error rate of the RF model, an accurate method for the identification of Chinese indigenous pig breeds was established.

## Figures and Tables

**Table 1:** Breeds and Sample Information.

Region	Breeds	Code	Number of Individuals
Cosmopolitan	Western Duroc	D	49
	Landrace	L	21
	Yorkshire	Y	53
	Pietrain	P	20
	Berkshire	B	16
Jiangsu, China	Small Meishan	SMS	75
	Mi	MI	36
	Erhualian	EH	42
	Huaibei	HB	34
	Hongdenglong	HD	30
	Jiangquhai	JQ	38
	Shan	SZ	20
Zhejiang, China	Bihu	BH	30
	Chunan	CA	59
	Chalu	CL	22
	Jinhualiangtouwu	JHL	57
	Lanxi	LX	40
	Shengxianhua	SH	64
	Jiangxing Black	JX	91
Shanghai, China	Middel Meishan	MMS	97
	Shawutou	SW	65
	Fengjing	FJ	32
	Pudong White	PD	68
Total	23 breeds	/	1059

## Data Availability

The original genotype files of 62,822 SNPs of 24 pig breeds can publicly obtained from the website (https://jbox.sjtu.edu.cn/l/uoaCjx, accessed on 6 June 2022). The LD-filtered 14,823 SNPs genotype file for the 23 pig breeds in this study are available in Supplementary Table S1.

## Supplementary Materials

The following supporting information can be downloaded at: https://www.mdpi.com/article/10.3390/genes13122207/s1. 1. Supplementary Table S1. Genotype file after LD pruning; 2. Supplementary Table S2. Top 1K importance SNP selected by MDA and MDG measures; 3. Supplementary Table S3. Classification effect of top 100SNPs panel selected by MDA measure.

## References

[1] Zhao Q.-b., Oyelami F.O., Qadri Q.R., Sun H., Xu Z., Wang Q.-S., Pan Y.-C. (2021). Identifying the unique characteristics of the Chinese indigenous pig breeds in the Yangtze River Delta region for precise conservation. BMC Genom..

[2] Bertolini F., Galimberti G., Calò D., Schiavo G., Matassino D., Fontanesi L. (2015). Combined use of principal component analysis and random forests identify population-informative single nucleotide polymorphisms: Application in cattle breeds. J. Anim. Breed. Genet..

[3] Gurgul A., Semik E., Pawlina K., Szmatoła T., Jasielczuk I., Bugno-Poniewierska M. (2014). The application of genome-wide SNP genotyping methods in studies on livestock genomes. J. Appl. Genet..

[4] Ferdosi M.H., Kinghorn B.P., Van der Werf J.H., Lee S.H., Gondro C. (2014). hsphase: An R package for pedigree reconstruction, detection of recombination events, phasing and imputation of half-sib family groups. BMC Bioinform..

[5] Brito L.F., McEwan J.C., Miller S.P., Pickering N.K., Bain W.E., Dodds K.G., Schenkel F.S., Clarke S.M. (2017). Genetic diversity of a New Zealand multi-breed sheep population and composite breeds’ history revealed by a high-density SNP chip. BMC Genet..

[6] Breiman L. (2001). Random forests. Mach. Learn..

[7] Liaw A., Wiener M. (2002). Classification and regression by randomForest. R News.

[8] Zhao Y., Fang L., Cui L., Bai S. (2020). Application of data mining for predicting hemodynamics instability during pheochromocytoma surgery. BMC Med. Inform. Decis. Mak..

[9] Chen X., Ishwaran H. (2012). Random forests for genomic data analysis. Genomics.

[10] Schiavo G., Bertolini F., Galimberti G., Bovo S., Dall’Olio S., Costa L.N., Gallo M., Fontanesi L. (2020). A machine learning approach for the identification of population-informative markers from high-throughput genotyping data: Application to several pig breeds. Animal.

[11] Browning B.L., Zhou Y., Browning S.R. (2018). A one-penny imputed genome from next-generation reference panels. Am. J. Hum. Genet..

[12] Zheng X., Levine D., Shen J., Gogarten S.M., Laurie C., Weir B.S. (2012). A high-performance computing toolset for relatedness and principal component analysis of SNP data. Bioinformatics.

[13] R Core Team (2013). R: A Language and Environment for Statistical Computing.

[14] Ren Y., Yu G., Shi C., Liu L., Guo Q., Han C., Zhang D., Zhang L., Liu B., Gao H. (2022). Majorbio Cloud: A one-stop, comprehensive bioinformatic platform for multiomics analyses. iMeta.

[15] Warr A., Affara N., Aken B., Beiki H., Bickhart D.M., Billis K., Chow W., Eory L., Finlayson H.A., Flicek P. (2020). An improved pig reference genome sequence to enable pig genetics and genomics research. Gigascience.

[16] Yin L., Zhang H., Tang Z., Xu J., Yin D., Zhang Z., Yuan X., Zhu M., Zhao S., Li X. (2021). rMVP: A memory-efficient, visualization-enhanced, and parallel-accelerated tool for genome-wide association study. Genom. Proteom. Bioinform..

[17] Ramos A., Megens H., Crooijmans R., Schook L., Groenen M. (2011). Identification of high utility SNPs for population assignment and traceability purposes in the pig using high-throughput sequencing. Anim. Genet..

[18] Wilkinson S., Wiener P., Archibald A.L., Law A., Schnabel R.D., McKay S.D., Taylor J.F., Ogden R. (2011). Evaluation of approaches for identifying population informative markers from high density SNP chips. BMC Genet..

[19] Lewis J., Abas Z., Dadousis C., Lykidis D., Paschou P., Drineas P. (2011). Tracing cattle breeds with principal components analysis ancestry informative SNPs. PLoS ONE.

[20] Seo D., Cho S., Manjula P., Choi N., Kim Y.-K., Koh Y.J., Lee S.H., Kim H.-Y., Lee J.H. (2021). Identification of Target Chicken Populations by Machine Learning Models Using the Minimum Number of SNPs. Animals.

[21] Hulsegge B., Calus M., Windig J., Hoving-Bolink A., Maurice-van Eijndhoven M., Hiemstra S. (2013). Selection of SNP from 50K and 777K arrays to predict breed of origin in cattle. J. Anim. Sci..

